# Inhibitory effect of topical Adelmidrol on antigen-induced skin wheal and mast cell behavior in a canine model of allergic dermatitis

**DOI:** 10.1186/1746-6148-8-230

**Published:** 2012-11-26

**Authors:** Santiago Cerrato, Pilar Brazis, Maria Federica della Valle, Alda Miolo, Anna Puigdemont

**Affiliations:** 1 UNIVET, Edificio Astrolabio, Avinguda Cerdanyola 92, 08172 Sant Cugat del Vallès, Barcelona, Spain; 2 Cedis, INNOVET Italia, Via Egadi 7, 20144, Milan, Italy; 3 Departament de Farmacología, Facultat de Veterinària, Universitat Autònoma de Barcelona, 08193, Bellaterra, Barcelona, Spain

**Keywords:** Adelmidrol, Aliamides, Mast cells, Allergy, Ascaris suum, Dogs

## Abstract

**Background:**

Adelmidrol is a semisynthetic derivative of azelaic acid and analogue of the anti-inflammatory compound palmitoylethanolamide (PEA). Based upon its physicochemical properties, adelmidrol is suitable for topical application. The main objective of the present study was to evaluate the efficacy of a topical adelmidrol emulsion on early and late inflammatory responses in hypersensitive dogs. Repeated intradermal injections of *Ascaris suum* extract were performed in both lateral thoracic areas of six conscious hypersensitive Beagle dogs, topically treated during 8 consecutive days. Adelmidrol (2%) was applied to one side and vehicle to the other. 24 hours after the last antigen challenge, two biopsies (adelmidrol- and vehicle-treated side) were obtained for each dog at the antigen injection site.

**Results:**

A significant reduction in the antigen-induced wheal areas was observed on the 4^th^ and 7^th^ day of adelmidrol treatment. Moreover, cutaneous mast cell numbers were significantly decreased in biopsies obtained after 8 consecutive days of topical adelmidrol treatment.

**Conclusions:**

The results obtained in the present study show that topical treatment with adelmidrol might represent a new therapeutic tool in controlling the early and late allergic inflammatory skin responses in companion animals.

## Background

Adelmidrol is the diethanolamide derivative of azelaic acid, i.e., naturally occurring dicarboxylic acid that has long proven to be an effective topical treatment for human inflammatory skin disorders [1], and whose mechanism of action has been extensively investigated [2]. Similar to the anti-inflammatory and anti-nociceptive compound palmitoylethanolamide (PEA) [3-12], adelmidrol belongs to the aliamide family [13,14], a group of fatty acid derivatives with cannabimimetic properties, able to control mast cell (MC) hyper-reactivity in several pathophysiological and pathological conditions [5,8,15-17]. We have recently found that PEA down-modulates the release of both preformed and newly-synthesized mediators from canine skin MCs challenged with immunologic stimuli [18]. Moreover, the PEA analogue adelmidrol has been shown to negatively control the behavior of canine skin MCs during pathophysiological conditions (i.e. healing of experimental wounds) [19]. In particular, a statistically significant increase of intracytoplasmic granular content of dermal MCs was shown in adelmidrol (2%)-treated wounds compared to control, thus suggesting the compound is effectively able to down-modulate skin MC degranulation in dogs [19]. Furthermore, the local application of adelmidrol confirmed the reduction in MC responses during chronic experimental inflammation, as shown by the significant decrease of mediators such as chymase which are selectively expressed by MCs and intimately involved in skin inflammation [20].

Mast cell hyperactivity is involved in the pathobiology of several canine disorders [21-23], including those of a dermatological nature [24,25]. In a canine model of allergic dermatitis, i.e., spontaneous hypersensitivity to the parasite *Ascaris suum*, the intradermal antigen exposure triggers the immediate degranulation of MCs, resulting in an early phase reaction (EPR), clinically manifested as skin wheals [24,26-30]. The combined actions of newly-synthesized and preformed mediators released by MCs results in the subsequent transcription of inflammatory cytokines and chemokines that ultimately drive the recruitment of inflammatory cells to the site of antigen injection. This process is known as the late phase reaction (LPR) and can become chronic [31,32]. We have recently found that a single oral dose of PEA (10 mg/kg) reduced significantly the antigen-induced skin wheal reaction in hypersensitive Beagle dogs [33]. Moreover, the repeated administration of PEA (5 and 10 mg/kg, intraperitoneal) has been shown to play a protective role against inflammation in experimental allergic dermatitis [34]. Unlike PEA, which is a highly lipophilic compound, adelmidrol is more suited to topical application, because exhibits both hydrophilic and lipophilic features (i.e,. amphipathic properties), which facilitates its absorbtion into the skin, whose epidermis is composed of alternating lipophilic and hydrophilic layers. A 4-week topical treatment with adelmidrol 2% emulsion in children affected by mild atopic dermatitis resulted in complete resolution in 80% of cases, with no side effects and no relapses at 8-week follow up [35]. In the last decades, the use of topical therapy in veterinary dermatology has increased and is especially recommended for localized allergic skin lesions, where it is currently regarded as a sole therapy or an adjunctive therapy minimizing the need for systemic treatments [36,37]. Based on the aforementioned background, the aim of the present study was to investigate whether, similar to PEA [33], adelmidrol was able to limit the inflammatory allergic response upon topical application.

## Methods

### Drugs, chemicals, and reagents

Adelmidrol (2%) and vehicle emulsions were purchased by Innovet (Milano, Italy). *A. suum* extract was purchased from Greer Laboratories (Lenoir, NC, United States). Evans blue dye and histamine diphosphate salt were obtained from Sigma-Aldrich (St. Louis, MD, United States).

### Animals

Six spontaneously hypersensitive Beagle dogs (four females and two males) with mean body weights of 15.2 ± 0.7 kg were used in this study. No drugs or additional treatments were given during the study, except sedatives, administered prior to obtaining the biopsies. All experiments and procedures were performed in accordance with European regulations governing the care and treatment of laboratory animals and were approved by the Animal Care and Use Committee of the Universitat Autònoma de Barcelona.

### Experimental protocol

**Figure 1:**
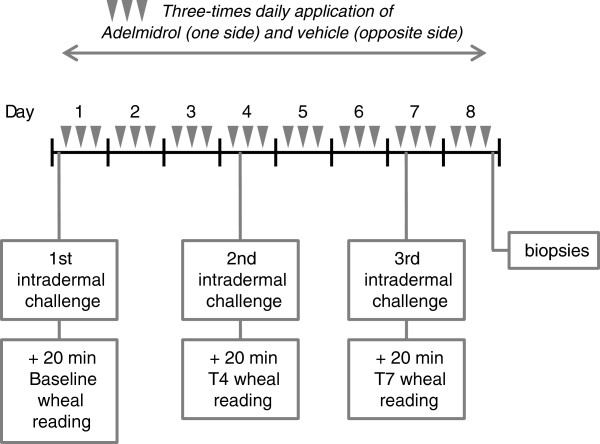
Schematic representation of the study timeline.

The method used was based on measurement of the inhibition of the wheal area induced, in the right and left shaved lateral thoracic areas (25 cm^2^ per area) of dogs, by intradermal injections of an *A*. *suum* extract solution (0.01% w/v), before and after treatments. Topical solutions (4 ml) of adelmidrol 2% and vehicle were applied three times a day for 8 consecutive days in the injected areas. In order to better visualize wheal areas, a 2% w/v Evans blue dye solution in saline was given intravenously (0.4 mL/kg) 30 min before each antigen injection. Three challenges were performed before (first challenge), on the 4^th^ day (second challenge) and the 7^th^ day (third challenge) of treatment, using a Hamilton syringe (type 701LT) (Reno, NV, United States). The results from the first challenge (i.e., performed prior to treatment) were taken as the baseline response. Each intradermal antigen challenge was performed 10 minutes before the second daily treatment application (Figure 1). Histamine (0.001% w/v) and saline were also injected, as positive and negative controls, respectively.

### Planimetry

10 minutes after intradermal challenge with *A*. *suum* extract solution, wheal areas were traced on an acetate sheet over the reaction site with indelible ink. Areas were then analyzed with an image analyzer MIP 4 ADVANCED (Digital Image System, Barcelona, Spain).

### Collection of skin biopsy specimens

24 hours after the third challenge, dogs received a local anaesthesia consisting of a subcutaneous injection of lidocaine (2%) without adrenaline, and two 6-mm punch skin biopsies (i.e., adelmidrol- and vehicle-treated side) were collected at injection sites.

### Histopathology

All biopsy specimens were fixed in neutral buffered formalin and embedded in paraffin for routine processing. 5 μm sections were stained with haematoxylin-eosin and toluidine blue (to visualize MC metachromatic granules). Dermis oedema and cell recruitment were graded semiquantitatively on an arbitrary scale [i.e., -/+ (0-1 cell); ++(1-3 cells); +++ (3-6 cells); ++++ (>6 cells)], on a x400 total magnification microscope. In particular, MC counts (per unit area) were determined on six randomly selected sections for each of the three different dermal layers, identified according to the known vascular plexuses subdivision in the skin, i.e., superficial (just beneath the epidermis), middle (around middle portions of hair follicles and sebaceous glands) and deep dermis (around the inferior portion of hair follicles and dermis/subcutis interface). Histological slides were read blindly with respect to treatment.

### Data analysis

To guarantee test reproducibility all experiments were performed by the same investigator blinded to the animal’s treatment. Wheal areas were expressed in mm^2^ as means ± standard error of the mean (mean ± SEM). Response variability in wheal areas was analyzed using the coefficient of variation (CV) before starting the treatments. Results were expressed as the percentage of inhibition observed for each challenge, comparing the antigen wheal area obtained in the same dog in the adelmidrol-treated side or vehicle-treated side versus the areas obtained at baseline (t_0_). Wheal inhibition was calculated with the following formula,

(1) $$\%\;\text{inhibition}=\frac{t_{n}\text{area}\;\left({\text{mm}}^{2}\right)-t_{0}\;\text{area}\;\left({\text{mm}}^{2}\right)}{t_{0}\text{area}\;\left({\text{mm}}^{2}\right)}\times 100$$

where t_0_ area corresponds to baseline value and t_n_ area is the area after adelmidrol or vehicle application, at a given time.

Differences between means were analyzed using an ANOVA test for paired measures and a Tukey’s multiple comparison post-hoc test. Statistical significance was set at *P*< 0.05 and *P*< 0.01.

Differences between MC numbers were compared using the Student’s *t*-test for paired data with a level of significance of *P*< 0.05 and *P*< 0.01.

Compliance with conditions for applying the aforementioned tests was verified with the Kolmogorov-Smirnov normality test.

## Results

### Macroscopic findings

In order to assess the repeatability of wheal area measure over time, wheal areas induced by subsequent *A*. *suum* extract injections in each dog were studied. Despite the differences observed between dogs (CV > 10%), no within-dog differences were observed (CV < 5%). For this reason, the adelmidrol inhibitory effect in each dog was calculated from its own basal value.

**Figure 2:**
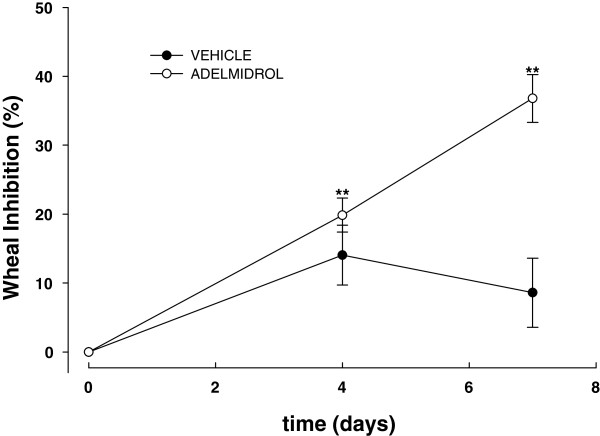
**Mean inhibition percentage (± SEM) (n = 6) of wheal areas induced in conscious hypersensitive Beagle dogs by*A.suum*extract observed before, on the 4**^th ^**and 7**^th ^**day of treatment with adelmidrol 2% or vehicle.** **P < 0.01.

Figure 2 shows the mean inhibition percentage of wheal areas induced in hypersensitive Beagle dogs by *A*. *suum* extract before and after 3 and 6 consecutive days of topical treatment with adelmidrol or vehicle, in comparison with baseline wheal areas. After 3 and 6 days of topical adelmidrol treatment, a statistically significant reduction in wheal areas was observed (*P* = 0.001 and *P* = 0.003), reaching an inhibition of 19.9 ± 2.5 and 36.8 ± 3.5%, respectively. The difference between the adelmidrol inhibitory effect observed on day 4 and 7 was statistically significant (*P* = 0.025). Conversely, the vehicle did not exert any significant effect on the wheal formation at any time (*P* = 0.054 and *P* = 0.3, at the two observation times respectively) (Figure 2).

### Microscopic findings

24 hours after the third intradermal allergen challenge (i.e., 8 days after starting treatment) a skin biopsy from each treatment side was obtained and the inflammatory reaction was histologically evaluated. The analysis revealed that oedema was evident within the vehicle-treated area in all six cases, while only two out of six dogs presented oedema in the adelmidrol-treated side. Furthermore, in the adelmidrol-treated side a slight, albeit not statistically significant, reduction in the recruitment of eosinophils, lymphocytes, monocytes and neutrophils was observed compared to the vehicle-treated side. Mast cell numbers decreased in almost all biopsies after treatment and for this reason a more accurate quantification was performed.

**Figure 3:**
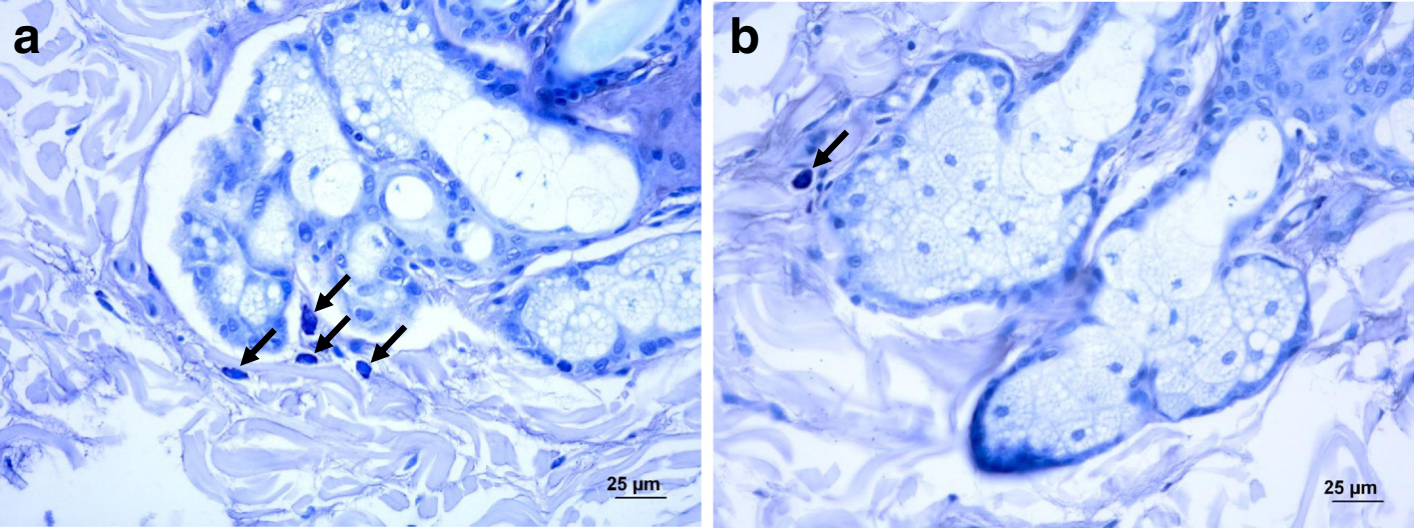
**Mast cells obtained from Beagle skin biopsies after toluidine blue staining (black arrows).** Histological sections from the vehicle-treated side (**a**) and adelmidrol-treated side (**b**).

Using toluidine blue staining for dermal MC counts, a highly significant reduction was observed in the adelmidrol-treated side (*P* = 0.0003) (Figure 3). Quantitative evaluation of MCs in the three different dermal layers that were studied (i.e., superficial, middle and deep dermis) revealed a significant decrease in MC numbers in the adelmidrol-treated side compared to the vehicle-treated side (Table 1).

**Table 1:** Quantification of mast cells (3 fields of 400X) in biopsies collected after topical vehicle and 2% adelmidrol treatment

**MAST CELL NUMBER OBSERVED AFTER VEHICLE AND ADELMIDROL TREATMENT**			
**Investigated dermal layer**	**ADELMIDROL**	**VEHICLE**	**P-VALUE**
	**(MEAN ± SEM)**	**(MEAN ± SEM)**	
Superficial	3.8 ± 0.6	6.6 ± 1.1	0.03
Middle	1.1 ± 0.3	2.9 ± 0.6	0.02
Deep	0.4 ± 0.2	1.3 ± 0.3	0.01

## Discussion

The present study shows, for the first time, that adelmidrol effects are evident in both the EPR and LPR in spontaneous hypersensitive Beagle dogs after intradermal antigen challenge. Topical treatment with adelmidrol (2%) for 3 and 6 consecutive days resulted in a significantly reduced skin wheal response compared to baseline values. Conversely, the vehicle-treated side did not show any significant wheal reduction at any treatment time, thus confirming, albeit indirectly, the lack of systemic absorption of adelmidrol after topical application.

### Magnitude of the inhibitory effect on skin wheal

Adelmidrol topical application inhibited wheal formation by about 20 and 37%, after 3 and 6 days of treatment, respectively. Results were of about the same magnitude as those observed in a previous study performed with a single oral dose of the aliamide parent molecule PEA in hypersensitive Beagle dogs [33]. The administration of PEA before intradermal antigen challenge resulted in a significant 29 and 32% reduction in wheal area, respectively, with doses of 10 and 30 mg/Kg [33].

A number of studies on the inhibitory effects of different drugs on skin wheal response in dogs have been published. The key findings are summarized in Table 2. The effect of corticosteroids (both oral or topical) on intradermally-induced skin wheal ranges from lack of effect [38,39] up to 45% inhibition [40-43], with the only exception of one single study (72% inhibition rate) [44]. Interestingly, similar results are observed in allergic human patients (i.e., 30-40% inhibition) [45], and better outcomes are known to occur with longer treatment [46]. The inhibitory effect of oral antihistamines on antigen-induced skin wheal in dogs is on average 50% [27,43], with obviously better results with histamine challenge [47,48] (Table 2). Interestingly, topically applied sodium cromoglycate (i.e., a MC stabilizer agent) inhibits the skin wheal in atopic human patients by only 27% [49].

**Table 2:** Main studies on the inhibition of skin wheal response in dogs - key findings

**Subjects**	**Intradermal challenge**	**Drug**	**Administration route**	**Treatment time**	**Wheal inhibition vs baseline**	**Ref**
Healthy Beagle dogs	Anti-canine IgE	Corticosteroids (prednisolone)	Oral	Twice daily for 3 days	No effect	[38]
Dogs with pruritic dermatitis	Histamine (5 different dilutions)	Corticosteroids (hydrocortisone 1%)	Topical (conditioner)	Twice weekly for 6 weeks	No effect	[39]
Healthy Beagle dogs	Histamine	Corticosteroids (betamethasone)	Topical (otic preparation)	Twice daily for 2 weeks	5.9%	[40]
Healthy Beagle dogs	D. farinae	Corticosteroids (betamethasone)	Topical (otic preparation)	Twice daily for 2 weeks	9%	[40]
Healthy Beagle dogs	Cynodon dactylon	Corticosteroids (betamethasone)	Topical (otic preparation)	Twice daily for 2 weeks	9.8%	[40]
Healthy Beagle dogs	Anti-canine IgE	Corticosteroids (hydrocortisone 1%)	Topical (conditioner)	3 days	14% (*)	[41]
Healthy dogs	Anti-canine IgE (different dilutions)	Corticosteroids (triamcinolone)	Topical (solution)	7 days	24-45% (§) depending on the stimulus concentration	[42]
Healthy mixed breed dogs	D. farinae	Corticosteroids (prednisone)	Oral	7 days	41%	[43]
Atopic Maltese-beagle cross-breed dogs	Anti-canine IgE	Corticosteroids (hydrocortisone aceponate)	Topical (spray)	7 days	72%	[44]
Healthy mixed breed dogs	D. farinae	Antihistamines (cetrizine)	Oral	14 days	49%	[43]
Spontaneously Ascaris hypersensitive Beagle dogs	A. suum	Antihistamines (rupatadine)	Oral	Single pre-treatment dose	35, 67 and 84% (#)	[27]
Spontaneously Ascaris hypersensitive Beagle dogs	A. suum	Antihistamines (loratadine)	Oral	Single pre-treatment dose	34, 61 and 66% (#)	[27]
Healthy Beagle dogs	PAF	Antihistamines (rupatadine)	Oral	Single pre-treatment dose	30% to 45% (¥)	[47]
Healthy Beagle dogs	Histamine	Antihistamines (rupatadine)	Oral	Single pre-treatment dose	50% to 80% (¥)	[47]
Healthy Beagle dogs	Histamine	Antihistamines (cetrizine, loratadine, rupatadine, levocabastine)	Oral	Single pre-treatment dose	70% to 80% (**)	[48]
Healthy Beagle dogs	Histamine	Antihistamines (cetrizine, loratadine, rupatadine, levocabastine)	Oral	Single pre-treatment dose	70% to 80% (**)	[48]

(*) versus placebo group;

(§) versus vehicle-treated side;

(#) peak activity at 0.1, 1 and 10 mg/Kg respectively, 2 to 4 hrs after administration;

(¥) peak activity, 4 h after administration, depending on the dose;

(**) peak activity, 4 h after administration, depending on the compound and the dose.

On the whole, one may consider wheal inhibition by topically applied adelmidrol to be almost the same order of magnitude as that of topical corticosteroids and lower than the value achieved after oral administration of antihistamines, whose clinical validity and utility remain doubtful [37]. Moreover, similarly to corticosteroids, treatment time could be a crucial factor [46] and longer treatment with topical adelmidrol (i.e., over 7 days) could increase the effect size.

### The purported mechanism involves mast cell control

Mast cells are considered to be major players of EPR [50-52]. Within minutes of antigen exposure, they rapidly secrete performed mediators (e.g., histamine, tryptase, chymase) and membrane-derived eicosanoids leading to the so-called ‘wheal and flare’ reaction of the skin [53,54]. The ability of aliamides (to whose family the tested compound belongs) to down-modulate the release of bioactive mediators from MCs [18-20] may thus represent the mechanism of the inhibitory effect on wheal areas, observed in the present study. Indeed, adelmidrol can down-modulate skin MC degranulation both during pathophysiological canine conditions [19] and in experimental chronic inflammation [20]. Moreover, the significant increase in the percentage inhibition of wheal area observed from the 4^th^ to 7^th^ day of treatment (from about 20 to 37% inhibition) may depend on the purported mechanism of action of the compound, i.e. the down-modulation of hyperactive skin MCs [19,20], which represents an endogenous tuning mechanism - and thus a gradual, progressive phenomenon - rather than an immediate ‘pharmacological’ switch-off effect [55].

The present study also demonstrates the inhibitory effect of adelmidrol on the increased MC number observed 24 h after the last antigen challenge (corresponding to the end of the 8-day-treatment). Indeed, the adelmidrol-treated side showed 42%, 62% and 69% less MCs in the superficial, middle and deep dermis respectively, compared to the vehicle-treated side. Although the baseline data are missing (the study was not designed to have t0 biopsies), this finding suggests that adelmidrol limited the LPR by decreasing skin MC hyperplasia following antigen challenge. Thus, both the functional and quantitative control of MCs might explain the observed anti-allergic effect of the topical treatment with adelmidrol. Indeed, similar findings emerged from a study on a model of chronic inflammation, where the local administration of adelmidrol both down-modulated MC degranulation and prevented numerical increase of MC numbers [20]. Interestingly, topical corticosteroids decrease MC numbers in humans [56], without any apparent effect on dogs [44].

Whether the effect on MC number observed in the present study depends on the inhibition of MC proliferation or maturation from progenitors, both resident and recruited from circulation, could not be tested by the techniques used in here. Both hypotheses may be possible, since the adelmidrol analogue PEA has been shown to (i) down-modulate keratinocyte production of the inflammatory cytokines [34], responsible for the recruitment of MCs [57]; and (ii) limit MC release of nerve growth factor [16], a neurotrophin that promotes chemotaxis of MCs as well as their maturation and degranulation [55,58,59]. Moreover, a recent study has shown the existence of an inhibitory “endocannabinoid tone” of skin MC biology, with skin MCs utilizing endocannabinoid signaling to limit not only their own activation/degranulation but also their maturation from resident progenitor cells [60]. This finding is particularly interesting since aliamides share several aspects of their biochemistry, metabolism and pharmacology with endocannabinoids and have been referred to as endocannabinoid-like, cannabimimetic compounds, or even indirect endocannabinoids [61,62]. Moreover, endocannabinoid receptors (i.e., CB1 and CB2) have been discovered in the canine skin and found to increase in diseased conditions, such as atopic dermatitis [63]. Finally, one should also consider that adelmidrol is a lipid amide and epidermal cells express the respective degrading enzymes (e.g., fatty acid amide hydrolase) [64]. Thus topically applied adelmidrol can be partially cleaved to release azelaic acid, which in turn might contribute to the observed effect, lessening the inflammatory phenotype of keratinocytes [2]. Importantly, no sign of irritation or any adverse effect was noticed during the present study.

### Practical considerations

From a practical point of view, the present findings suggest that adelmidrol could find a place in the management of hypersensitive skin disorders in the dog, where topical treatments are currently preferred to systemic ones for localized lesions characterizing the less severe stages of the disease. Alternatively, adelmidrol could be applied as an adjunct to systemic treatments for a “dual-site action” in selected cases, as suggested for more classical topical tools [37]. The most frequently used topical treatments for canine allergic skin diseases are glucocorticoid formulations and tacrolimus oinment. Both approaches are effective, even though tacrolimus has a slow onset of action, and both exert adverse effects (i.e., mild irritation, cutaneous atrophy, superficial follicular cysts, and increased susceptibility to skin infection) that, although mild, can prejudice owner compliance and lengthen recovery [37].

## Conclusions

Intradermal injection of allergic-inflammatory stimuli, with measurement of immediate wheal reaction and later dermal cellular infiltrates has been considered a useful technique for objectively documenting the anti-inflammatory effect of topical preparations in dogs [42]. The present study evaluated the effect of the topical daily application of the aliamide adelmidrol on the canine skin response to intradermal allergen challenge and confirmed its anti-inflammatory effect. In particular, the tested aliamide significantly reduced both EPR and LPR in hypersensitive dogs. A significant decrease in acute inflammation response and MC numbers was observed, without any sign of irritation or adverse effect. Even though further and more detailed molecular studies are needed to confirm the results and broaden knowledge on the mechanisms involved, the present findings suggest that adelmidrol emulsion could represent a valuable and safe tool in the armamentarium for canine inflammatory allergic skin disorders. The effects on wheal and MC numbers provide a sound basis for an anti-inflammatory drug sparing effect, and predict adelmidrol to be profitably combined with oral treatments for an optimal dual site therapy, i.e. outside-inside approach, currently the most preferred route for managing canine hypersensitive skin disorders [37].

## Competing interests

Alda Miolo is an employee of CeDIS (Science Information and Documentation Centre), Innovet Italia srl. Maria Federica della Valle is a scientific consultant for the same company. None of the other authors declare a conflict of interest.

## Authors’ contributions

SC and AP carried out the experiments and analyzed the data. AM and MFdV participated in the design of the study and helped to draft the manuscript. AP and PB conceived of the study, and participated in its design and coordination and helped to draft the manuscript. All authors read and approved the final manuscript.
